# In-Depth Analysis of Human Neonatal and Adult IgM Antibody Repertoires

**DOI:** 10.3389/fimmu.2018.00128

**Published:** 2018-02-05

**Authors:** Binbin Hong, Yanling Wu, Wei Li, Xun Wang, Yumei Wen, Shibo Jiang, Dimiter S. Dimitrov, Tianlei Ying

**Affiliations:** ^1^Key Laboratory of Medical Molecular Virology of Ministries of Education and Health, School of Basic Medical Sciences, Fudan University, Shanghai, China; ^2^Central Laboratory, The Second Affiliated Hospital of Fujian Medical University, Quanzhou, China; ^3^Protein Interactions Section, Cancer and Inflammation Program, Center for Cancer Research, National Cancer Institute, National Institutes of Health, Frederick, MD, United States; ^4^Shanghai Blood Center, WHO Collaborating Center for Blood Transfusion Services, Shanghai, China

**Keywords:** high-throughput sequencing, antibody repertoire, cord blood, VDJ rearrangement, junctional modification, N2 addition

## Abstract

Although high-throughput sequencing and associated bioinformatics technologies have enabled the in-depth, sequence-based characterization of human immune repertoires, only a few studies on a relatively small number of sequences explored the characteristics of antibody repertoires in neonates, with contradictory conclusions. To gain a more comprehensive understanding of the human IgM antibody repertoire, we performed Illumina sequencing and IMGT/HighV-QUEST analysis of IgM heavy chain repertoire of the B lymphocytes from the cord blood (CB) of neonates, as well as the repertoire from peripheral blood of healthy human adults (HH). The comparative study revealed unexpectedly high levels of similarity between the neonatal and adult repertoires. In both repertoires, the VDJ gene usage showed no significant difference, and the most frequently used VDJ gene was IGHV4-59, IGHD3-10, and IGHJ3. The average amino acid (aa) length of CDR1 (CB: 8.5, HH: 8.4) and CDR2 (CB: 7.6, HH: 7.5), as well as the aa composition and the average hydrophobicity of the CDR3 demonstrated no significant difference between the two repertories. However, the average aa length of CDR3 was longer in the HH repertoire than the CB repertoire (CB: 14.5, HH: 15.5). Besides, the frequencies of aa mutations in CDR1 (CB: 19.33%, HH: 25.84%) and CDR2 (CB: 9.26%, HH: 17.82%) were higher in the HH repertoire compared to the CB repertoire. Interestingly, the most prominent difference between the two repertoires was the occurrence of N2 addition (CB: 64.87%, HH: 85.69%), a process that occurs during V-D-J recombination for introducing random nucleotide additions between D- and J-gene segments. The antibody repertoire of healthy adults was more diverse than that of neonates largely due to the higher occurrence of N2 addition. These findings may lead to a better understanding of antibody development and evolution pathways and may have potential practical value for facilitating the generation of more effective antibody therapeutics and vaccines.

## Introduction

High-throughput sequencing of antibody repertoire and related bioinformatics analysis are becoming increasingly important tools that allow unprecedented insight into the in-depth, sequence-based composition of human immune repertoires (1–3). Such information may lead to a better understanding of antibody development and evolution pathways and facilitate the generation of more effective antibody therapeutics and vaccines (4, 5). However, despite the extensive efforts over the past decade, our understanding of human antibody repertoire remains limited due to its two fundamental characteristics. First, the antibody repertoire of an individual is highly dynamic, which varies greatly not only in response to environmental (for example, infection) but also to intrinsic (for example, aging) factors. Furthermore, a thorough analysis of antibody repertoire has been hindered by its enormous diversity. There are three primary mechanisms contributing to the antibody repertoire diversity: the combinatorial diversity created by rearrangements of the variable (V), diversity (D), and joining (J) gene segments; the junctional diversity resulted from exonuclease trimmings and the random addition of nucleotides; and the somatic hypermutations that occur during the immunoglobulin synthesis. By these mechanisms, a virtually unlimited number of different antibodies could be achieved using a limited number of germline immunoglobulin genes (6, 7). Therefore, it could be technically challenging to analyze the highly dynamic and diverse human antibody repertoires.

Notably, the antibody repertoire of the fetus or umbilical cord blood (CB) represents a source of un-mutated or minimally mutated antibodies, thus providing a unique opportunity to gain a general understanding of the human antibody repertoire. Compared with adults, human neonates are believed to have a limited ability to generate effective antibodies because they have not been exposed to exogenous antigens and do not develop an effective immunological memory response to the antigens (8, 9). Accordingly, the fetal repertoire is more restricted than the adult repertoire (10). Several earlier studies have revealed the characteristics of the fetal and adult immune repertoires, including the preferential VDJ gene usages, somatic mutations, and the length of the CDR3, which vary in different periods of human fetal life and adulthood (11–14). In contrast, another study compared the repertoires of human CB and adult sources reconstituted by NOD/SCID/β2m^−/−^ mice with human B-cell progenitors and found nearly identical IGHV and IGHJ gene segment usage and only modest differences in CDR3 of the antibody heavy chain (15). Such inconsistency may partly result from the relatively small size of the examined samples or the sequenced repertoires. Indeed, we estimate that there are at least 10^7^ B lymphocytes in a typical CB sample (100–200 mL), whereas the 454 pyrosequencing technology, as used in most previous studies, was only able to generate roughly 10^5^ reads per sample (16), and thus underpowered to evaluate the full scale of antibody repertoires.

Recently, the Illumina-based sequencing is becoming the dominant high-throughput sequencing strategy, enabling the acquisition of millions to billions of sequences in a single experiment. The greater sequencing depth allows comprehensive investigation of the human antibody repertoires with high diversity (17). In this study, we described the characterization of the IgM heavy chain (IgH) repertoires from the B lymphocytes of the CB of neonates and the peripheral blood of adults using the Illumina sequencing platform. Over 10^7^ unique antibody clones were identified, but less than 1% of these unique clones were shared by both neonates and adults, indicating the extremely large diversity of human antibody repertoires. Interestingly, despite the difference in sequences, we found unexpectedly high levels of similarity between the neonatal and adult repertoires regarding the VDJ gene usage, the characteristics of CDRs, and the occurrence of certain junctional modifications. The IgH repertoire of healthy adults was more diverse than that of neonates, largely due to the higher occurrence of N2 addition, a process that occurs during V-D-J recombination for introducing random nucleotide additions between D- and J-gene segments. These findings suggest a critical and previously unrecognized role for antibody junctional modifications, especially N2 addition, in the development and evolution of antibody repertoires in healthy individuals.

## Materials and Methods

### Samples

The CB samples from 10 newborn babies (4 boys and 6 girls) were provided by National Disease Research Interchange (NDRI, Philadelphia, PA, USA) with approval of institutional research board and donor consent. Care was taken not to contaminate the samples with maternal blood. The blood samples from healthy adults were collected from 33 healthy adults (16 females and 17 males; age range, 27–62 years; average age, 44.1 years), who underwent a routine health check with no history of known major diseases, with approval of institutional research board and donor consent. The basic characteristics of the study population were summarized in Table 1, and the detailed inclusion criteria were summarized in the Supplementary Materials.

**Table 1:** Characteristics of the study population.

	Neonates	Adults
Gender (F/M)	6/4	16/17
Age	0 day	F: 43.8 ± 9.9 (years)M: 45.0 ± 9.4 (years)
Weight	3,379.6 ± 561.8 (g)	N/A

*F, female; M, male; N/A, not available*.

### Establishment of IGH Repertoires for Deep Sequencing

As the source for amplification of antibody sequences, cDNA was reverse transcripted from the total RNA extracted from lymphocytes and was prepared according to the reported protocols (18). PCR amplifications were applied to establish the IGH repertoire libraries. Primers used in PCR amplifications were highly specific to the N-terminal and C-terminal regions of the IgM-derived heavy chains as described previously (19). Briefly, PCR amplifications were performed with a mixture of primers in which the 3′-ends ligated to the first seven codons of IGHV1 to IGHV7 gene families, and PCR amplifications of the constant domains were performed by a sense primer specific for CH1 domain of IGHM spanning first eight codons (3′–5′ strand) according to the ImMunoGeneTics database (www.imgt.org). The PCR amplifications were performed again to produce shorter IgM fragments for Illumina sequencing. Multiplexed PCR was employed to amply rearranged IGH sequences using forward primers matching the first frame regions in IGHV gene segments and reverse primers aligning the fourth frame regions in IGHJ gene segments, which covered the antibody variable domains consisting of the three CDRs. The primers used in our study were listed in the Supplementary Materials. PCR amplification were performed in a volume of 50 µL, using 25 µL Pfu mastermix (CWbio, China), 1 µL template, and 1 µL (50 nM) each primer mixture. The PCR conditions were as follows: initial denaturation at 94°C for 5 min, 35 cycles of denaturation at 94°C for 30 s, annealing at 56°C for 1 min, extension at 72°C for 1 min, and final extension at 72°C for 10 min. The PCR amplicons were purified using the QIAquick Gel Extraction Kit (Qiagen, Germany), then underwent high-throughput sequencing based on Illumina Hiseq platform according to the manufacturer’s protocol.

### Sequences Processing

A series of stringent quality control criteria were applied to exclude biologically implausible sequences. First, raw reads were filtered for Phred quality score of 20 over 80% of nucleotides to gain clean data to exclude sequences with PCR errors and sequencing artifacts. The sequences were classified to productive and unproductive groups according to the analysis of IMGT/HighV-QUEST. The unproductive VDJ rearrangements were eliminated from the dataset, and the productive sequences were excluded when containing insertions and deletions (indels) or stop codons in V- and J-gene segments. These indels or stop codons would break the reading frames in VDJ segments. It is believed that the B cells need a functional antigen receptor to survive (20), and therefore, when such breaks appeared, sequences might contain either PCR errors or sequencing artifacts (21). Furthermore, sequences carrying substitutions or mutations in the conserved amino acids at specified positions were removed to avoid the substitution errors in Illumina platform. The possibility of misclassification of VDJ gene segments in the algorithms of the IMGT tool for VDJ region searching mainly depended on these special amino acids (22). Additionally, the redundant sequences were eliminated to avoid the accumulation of one single sequence due to PCR amplification. The unique clones were defined by sequences containing unique VDJ, including unique alleles and CDR3 sequences. The number of sequences after each step of sorting is listed in Table 2. The sequences have been deposited in the NCBI SRA database (SUB3220644). IMGT/High V-QUEST (version 1.5.1) was used for sequence annotation to determine the V(D)J genes, CDRs, and junctional modification and to identify indels errors (23). Results from IMGT/High V-QUEST analysis were imported into PostgreSQL database, and Structured Query Language (SQL) was used to retrieve the data for statistical analysis.

**Table 2:** The number of input cells and sequencing data.

	Neonates	Adults
Theoretical number of lymphocytes^a^	1.1–2.1 × 10^9^	6–9.6 × 10^9^
Input cells (10^8^/100 mL)	1.0 × 10^9^	6.6 × 10^9^
Raw sequences (clean data)	10,122,711 (1% of input cells)	15,978,350 (0.24% of input cells)
Unique sequences (nt)	8,475,193	15,057,048
Productive sequences	6,532,659 (77.0% of unique sequences)	11,820,648 (78.5% of unique sequences)
Unproductive sequences	428,411 (5.1% of unique sequences)	629,333 (4.2% of unique sequences)
Error sequences	1,514,123 (17.9% of unique sequences)	2,607,067 (17.3% of unique sequences)
Unique clones (productive)	3,209,817	7,303,188

*^a^The theoretical number of lymphocytes was estimated by the estimated number of lymphocytes reported previously (0.5–0.9 × 10^9^ cells/L in neonates and 0.16–0.68 × 10^9^ cells/L in adults, Table S1 in Supplementary Materials)*.

### Statistical Analysis

Data analyses were performed using GraphPad Prism, Perl, and R programs. Student’s *t*-test, Pearson’s chi-test, and logistic regression analyses were used in the statistical significance analyses when required. Because statistically significant differences are more likely to occur with large sample sizes, effect sizes are necessary to understand if the differences are meaningful. The effect size of Student’s *t*-test is Cohen’s *d* value, which is used to measure the standardized difference between two means, as initially suggested by Cohen (24): when *d* = 0.20, the ES or the difference is considered to be small; when *d* = 0.50, the ES is medium; and when *d* = 0.80, the ES is large. For chi-square analyses and logistic regression, the odds ratio (OR) was used as the effect size (25). Generally, OR values that ranged from 0.9 to 1.1 was considered to be not significantly different; when 1.2 < OR < 1.4 or 0.7 < OR < 0.8, the difference was slight; when 1.5 < OR < 2.9 or 0.4 < OR < 0.6, the difference was medium; and when OR > 3.0 or OR < 0.3, the difference was large or the association was strong.

### Ethics Statement

The CB samples were provided by NDRI (Philadelphia, PA, USA) with approval of the institutional research board and the donor consent. Procedures followed in this study were in accordance with the ethical standards of concerned institutional policies and the Research Donor Program of National Cancer Institute.

## Results

### The Repertoire Diversity

**Figure 1:**
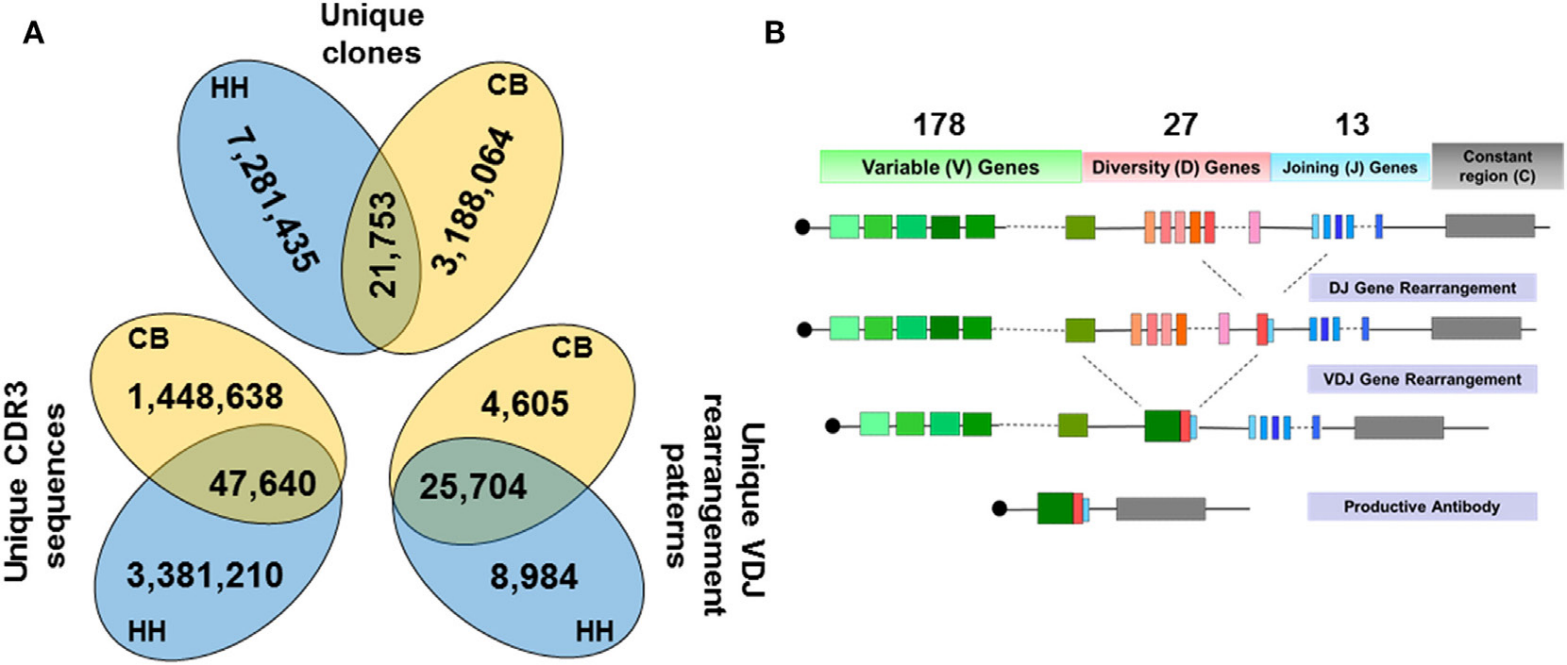
**(A)** Data summary of the unique clones, the unique CDR3 sequences, and the unique VDJ gene rearrangement patterns from the repertoires of the neonates and adults. **(B)** Schematic of VDJ gene rearrangement, showing the number of V, D, and J gene (including alleles) observed in our dataset.

By high-throughput sequencing, we obtained two immune repertoires of IgHs, one from the B cells in the CB of healthy neonates (CB), and the other from the B cells in peripheral blood of healthy human adults (HH). Initially, 10,122,711 raw sequences were collected from CB, and 15,978,350 sequences were obtained from HH. Next, we performed a series of stringent data filtering and cleaning procedures to exclude unproductive or biologically implausible sequences, as described under the Section “Materials and Methods.” The sequences that had unique VDJ gene rearrangements, including those contained unique CDR3 amino acid (aa) sequences, or had identical CDR3 but distinct VDJ rearrangements were defined as “unique clones.” A total of 3,209,817 unique clones (31.7% of raw sequences) were identified in the CB repertoire, and 7,303,188 unique clones (45.7% of raw sequences) were found in the HH repertoire (Figure 1A). To exclude the bias caused by the number of input cells or the sequences, we randomly selected sequences from each datasets using the randomized table generated by R program (repeated three times), which represents the computational simulation to sample the same amount of input cells or sequences. Then, we calculated the proportion of the unique clones out of the randomly selected sequences. The results showed that, when the sample size was small, the proportion of the unique clones did not differ greatly between CB and HH. In contrast, when the sample size increase to 1,000,000, the proportion of the unique clones began to show difference between CB and HH (CB: 66%, HH 77%), indicating that the HH repertoire was more diverse than CB (Figure S1 in Supplementary Material).

Interestingly, we found that the HH and CB repertoires only shared 21,753 unique clones, constituting 0.7% of the CB and 0.3% of the HH unique clones, respectively (Figure 1A). Among these unique clones, 1,496,278 unique CDR3 aa sequences (46.6% of unique clones) were identified in the CB repertoire, and 3,428,850 unique CDR3 (46.7% of unique clones) were found in the HH repertoire (Figure 1A). Similarly, only 47,640 CDR3 sequences were shared by both repertoires, constituting 3.2% of the CB and 1.4% of the HH unique CDR3 sequences.

Next, we analyzed the VDJ rearrangement patterns using the IMGT/High V-QUEST tool (version 1.5.1). We included the gene allele information in the calculation of VDJ gene patterns to estimate the antibody repertoire diversity, because the allele information represents the genetic polymorphism that also results in repertoire diversity. There were 30,309 and 34,688 unique VDJ patterns in CB and HH repertoires, respectively, rearranged by 178 germline V-, 27 D-, and 13 J-gene segments (Figures 1A,B). The two repertoires shared 25,704 identical VDJ rearrangement patterns, which accounted for 84.8% of patterns in CB repertoire and 74.1% in HH repertoire. Taken together, these results highlight the overwhelmingly high diversity of human IgH repertoires as little antibody sequences were shared by two different repertoires, although recombined from similar VDJ genes and rearrangement patterns.

### VDJ Gene Usage

**Figure 2:**
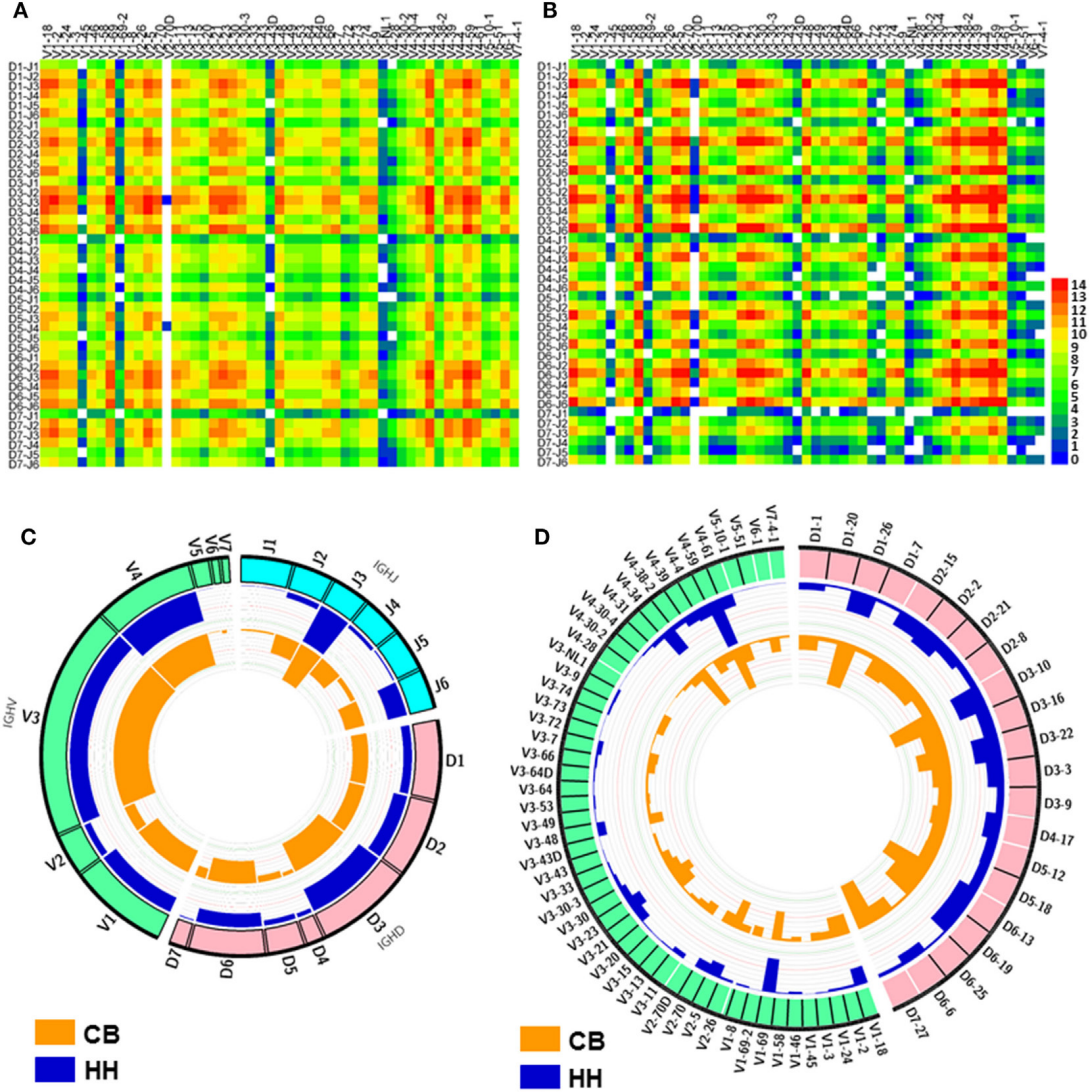
The VDJ gene rearrangements and VDJ gene usage observed in the repertoires of the neonates and adults. **(A,B)** The heatmap of VDJ gene rearrangements observed in the IgH repertoire of the neonates **(A)** and the adults **(B)**, The base-2 logarithm of the count was used to present the frequency of each type of rearrangements. **(C)** The usage of VDJ gene families. The outsider arcs represent the V (green arc), D (pink arc), and J (cyan arc) gene families, and the histograms inside the circle represent the usage of each gene in the repertoire of the neonates (orange) and adults (blue). **(D)** The usage of V (green arc) and D (pink arc) gene subgroups in the repertoire of the neonates and adults. The histograms inside the circle represent the usage of each gene subgroups in the repertoire of the neonates (orange) and adults (blue).

To find the preferentially utilized VDJ gene in the two repertoires, the usages of the IGHV, IGHD, and IGHJ gene segments were calculated and shown in Figure 2. In the VDJ gene usage analyses, the gene alleles were not included to pack the data and reduce the data groups. There are 51 IGHV genes belonged to 7 gene families (Figures 2A,B). The top three preferred IGHV genes were IGHV4-59 > IGHV4-34 > IGHV2-5 in the CB repertoire (Figures 2A,D), and were IGHV4-59 > IGHV1-69 > IGHV4-34 in the HH repertoire (Figures 2B,D). Of all seven IGHV gene families, IGHV1, IGHV2, IGHV3, and IGHV4 gene families were mainly used, and together accounted for 94.5% in CB repertoire and 99.9% in HH repertoire (Figure 2C). On the other hand, a dramatic decreased use of IGHV5, IGHV6, and IGHV7 gene families was found in the HH repertoire as compared to that in the CB (0.1% vs. 5.5%). The usage of IGHV4 gene family (43.5%) was much higher than the other gene families in the HH repertoire, while both IGHV3 and IGHV4 gene families were frequently observed in the CB repertoire, with a rate about 30% (Figure 2C).

In both repertoires, the most populated group in the IGHD sets was IGHD3, with a rate of 28.7% in CB and 34.1% in HH (Figure 2C). IGHD7 or IGHD7-27, the only member in IGHD7 family, was rarely observed in the HH repertoire (1.3%) but accounted for about 10% in CB. The detailed classifications of IGHD gene groups revealed the top three frequently used IGHD genes: IGHD3-10 > IGHD6-13 > IGHD7-27 in CB and IGHD3-10 > IGHD3-22 > IGHD1-26 in the HH repertoire (Figure 2D).

For IGHJ genes, the usage of IGHJ2, IGHJ3, IGHJ4, and IGHJ6 accounted for a large proportion (more than 90%), while IGHJ1 and IGHJ5 were comparatively used less in both repertoires, with a rate of 6.9% in CB and 2.0% in the HH repertoire. IGHJ3 was the most frequently used group in both repertoires, with a rate of 37.4% in CB and 57.3% in HH.

Although the comparison of VDJ gene usage between the two repertoires showed that the difference was statistically significant (*p* < 0.05, Table 3), the OR values were close to 1.0, indicating that the effect was small, and the low *p* value was mainly due to the large sample size. Indeed, as shown in Figure 2, the difference was only slight between the two repertoires. In both repertoires, the most frequently used VDJ gene was IGHV4-59, IGHD3-10, and IGHJ3. Taken together, these results suggest that the VDJ gene usage was similar in the IgH repertoire of neonates and adults.

**Table 3:** The VDJ gene usage distribution in the repertoires of neonates and adults.

Gene group	Repertoire		*p*^b^	OR^b^	95% CI^b^
	CB^a^ %	HH^a^ %			
IGHV1	22.08	22.05	2.27E−58	1.009	(1.008, 1.010)
IGHV2	10.39	9.19			
IGHV3	32.53	25.16			
IGHV4	30.42	43.47			
IGHV5	0.86	0.10			
IGHV6	3.27	0.02			
IGHV7	0.45	0.02			

IGHD1	14.78	12.07	<2.2E−16	0.894	(0.893, 0.894)
IGHD2	14.93	19.94			
IGHD3	28.72	34.10			
IGHD4	3.86	5.89			
IGHD5	6.47	7.40			
IGHD6	21.23	19.33			
IGHD7	10.01	1.29			

IGHJ1	1.98	0.38	<2.2E−16	1.113	(1.112, 1.114)
IGHJ2	18.36	8.01			
IGHJ3	37.44	57.25			
IGHJ4	18.83	4.97			
IGHJ5	4.93	1.64			
IGHJ6	18.48	27.74			

*^a^CB: the repertoire of the neonates; HH: the repertoire of the adults*.

*^b^Calculated by the logistic regression*.

*OR, odds ratio; 95% CI, 95% confidence interval*.

### The Characteristics of CDRs

**Figure 3:**
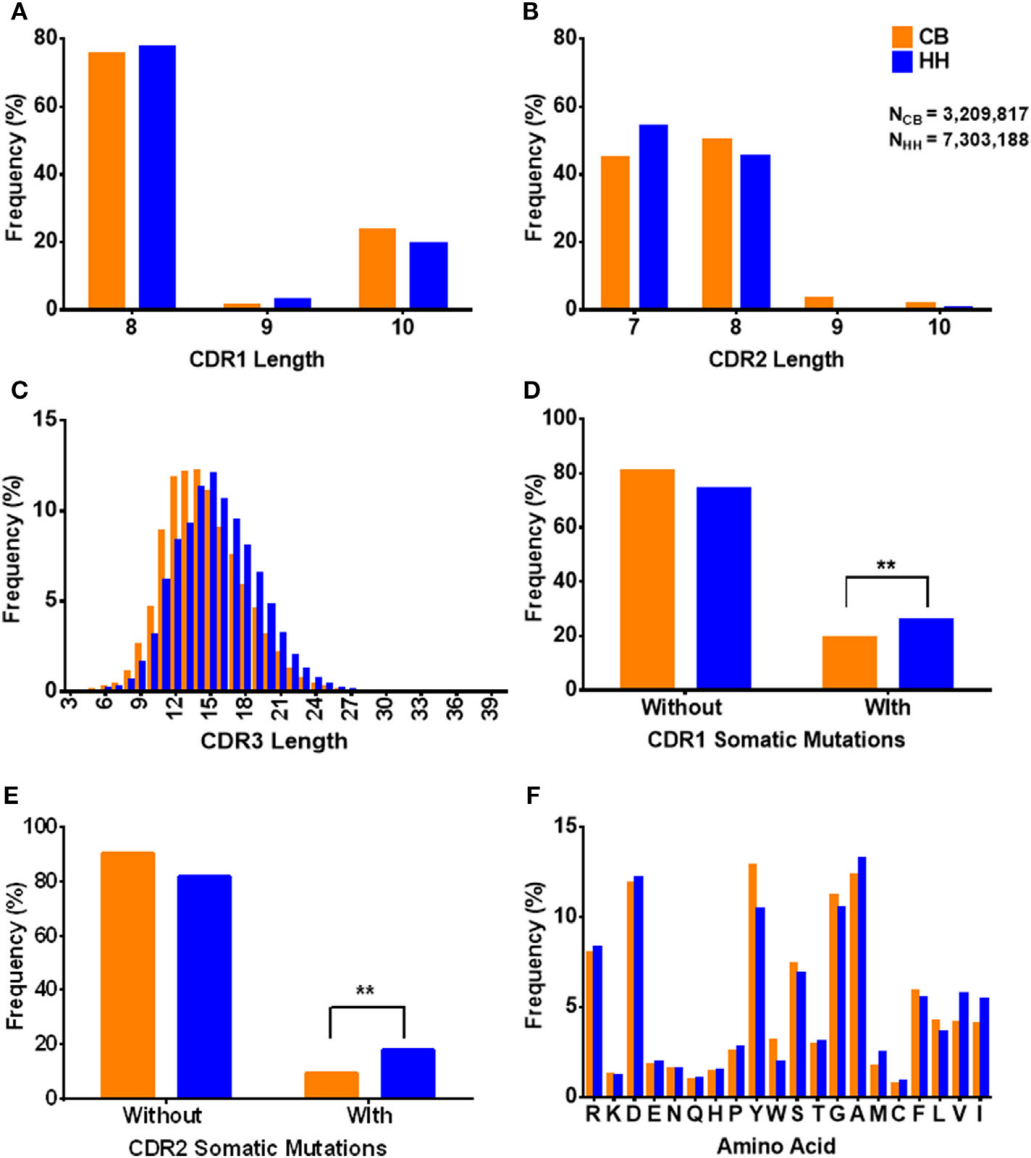
The characteristics of CDRs in the IgH repertoire of the neonates and the adults. **(A)** The CDR1 length distribution. **(B)** The CDR2 length distribution. **(C)** The CDR3 length distribution. **(D)** The occurrence of amino acids changes in CDR1 regions. “Without” represented no amino acids changes, and “With” represented at less one amino acid change happened when compared to the germline IgH gene. **(E)** The occurrence of amino acids changes in CDR2 regions. **(F)** The amino acids usage of CDR3 sequences.

The CDRs play critical roles in the binding of antibodies to antigens. In both repertoires, the length of CDR1 ranged from 8 to 10 aa (Figure 3A) and CDR2 ranged from 7 to 10 aa (Figure 3B). The CDR1 length of 8 aa was the most common observed (CB: 75.38%, HH: 77.63%), and the CDR2 length of 7 aa and 8 aa together accounted for the majority of the repertoires (CB: 94.91%, HH: 99.56%). As shown in Table 4, there was no apparent difference in the average length of CDR1 (CB: 8.5, HH: 8.4) or CDR2 (CB: 7.6, HH: 7.5) between the CB and the HH repertoires.

**Table 4:** The average length (aa) of CDRs in the repertoires of neonates and adults.

CDR	Repertoire		*T*^a^	*d*	*p*	95% CI
	CB	HH				
CDR1	8.48 ± 0.85	8.42 ± 0.8	108.443	0.07	<2.2E−16	(0.059, 0.062)
CDR2	7.62 ± 0.64	7.47 ± 0.52	381.801	0.28	<2.2E−16	(0.154, 0.156)
CDR3	14.51 ± 3.33	15.48 ± 3.43	−432.565	0.29	<2.2E−16	(−0.978, −0.969)

*CDR, the complementarity-determining region*.

*^a^Calculated by Student’s t-test*.

Interestingly, we found that the CDR3 length in the HH repertoire was evidently longer than the CB repertoire. Compared to that of CDR1 and CDR2, the length of CDR3 was much more variable, ranged from 3 to 42 aa in the CB repertoire and from 3 to 38 aa in the HH repertoire. As shown in Figure 3C, the CDR3 length of 14 aa was the most frequently observed in the CB repertoire, while the 15 aa CDR3 accounted for the largest proportion in the HH repertoire. Furthermore, for CDR3 length of 14 aa or smaller, the CB repertoire exhibited significantly higher frequencies, but just the opposite for CDR3 length of 15 aa or larger.

The aa changes in CDR1 and CDR2 as compared to germline sequences were also calculated in our analysis. The proportion of sequences with aa changes in CDR1 region was 19.33% in the CB repertoire and 25.84% in the HH repertoire (Figure 3D). Logistic regression showed that the rate of aa changes in CDR1 region of the HH repertoire was about 1.5 times higher than the CB [OR = 1.454, 95% CI: (1.449, 1.459)]. Similarly, the proportion of sequences with aa changes in CDR2 region was 9.26% in the CB repertoire and 17.82% in HH (Figure 3E), and the rate of aa changes in CDR2 region of HH was about twice as much as that of the CB repertoire as defined by logistic regression [OR = 2.124, 95% CI: (2.115, 2.133)]. As expected, these results indicate that the extent of somatic hypermutation occurred in the CDR1 and CDR2 regions was higher in the IgH repertoire of adults than that of the neonates.

The aa changes in CDR3 region cannot be calculated due to the extremely high flexibility of this region. Therefore, the aa usage of CDR3 region was analyzed instead, as shown in Figure 3F. Tyrosine, alanine, glycine, and aspartic acid were the most frequently occurring amino acids in CDR3. The aa composition of the CDR3 demonstrated no significant difference between the two repertories [OR = 0.993, 95% CI: (0.993, 0.993)]. The hydrophobicity value of the amino acids was determined by Kyte-Doolittle scale. The average hydrophobicity value of all the CDR3 sequences was −0.43 ± 2.70 for the CB repertoire and −0.28 ± 2.82 for HH and showed no significant difference [*t*-value = −296.29, Cohen’s *d* = 0.05, *p* < 2.2E−16, 95% CI: (−0.144, −0.141)]. Taken together, these results suggest that the CDRs in the IgH repertoire of adults have characteristics similar to that of neonates, except for the slightly higher level of somatic hypermutation and significantly longer CDR3 regions.

### V-D-J Junction Analysis

**Figure 4:**
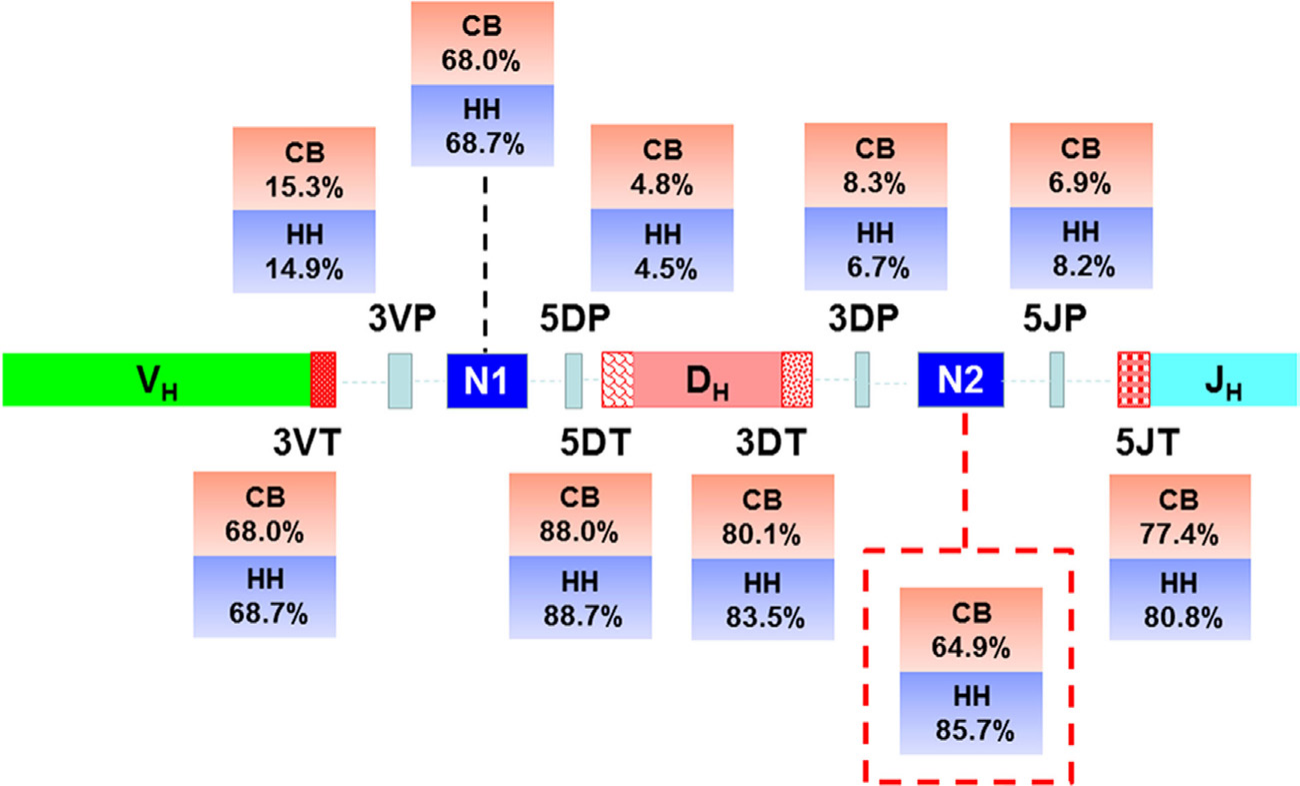
Schematic of the junctional modifications during VDJ rearrangement, showing the locations and the occurrence of different types of junctional modifications. 3VP and 3VT: the palindromic nucleotides (P) additions and exonuclease trimmings observed at 3′-end of V regions, respectively; 5DP and 5DT: the P additions and exonuclease trimmings observed at 5′-end of D genes, respectively; 3DP and 3DT: the P additions and exonuclease trimmings observed at 3′-end of D genes, respectively; 5JP and 5JT: the P additions and exonuclease trimmings observed at 5′-end of J genes, respectively; N1: the non-template randomized nucleotides (N) additions happened at the region between the 3′-end of V gene and the 5′-end of D gene; N2: N additions happened at the region between the 3′-end of D gene and the 5′-end of J gene.

In addition to recombinational diversity, the diversity of IgH repertoire also came from the V-D-J junctions including the palindromic nucleotides (P) addition and the non-template randomized nucleotides (N) addition, as well as the deletion of nucleotides caused by exonuclease trimming (T). The N additions happened at the region between the 3′-end of V gene and the 5′-end of D gene (N1) and the region between the 3′-end of D gene and the 5′-end of J gene (N2). The P additions and the exonuclease trimming were observed at 3′-end of V regions (3VP and 3VT), 5′-end and 3′-end of D genes (5DP and 5DT, 3DP and 3DT) and 5′-end of J genes (5JP and 5JT). The occurrence of the P/N addition and exonuclease trimming is shown in Figure 4. Notably, the occurrence of N2 addition were significantly higher in the HH repertoire than CB, and the occurrence of 3DP and 3DT, 5JP, and 5JT showed slight difference between the two repertoires, while other types of modification showed no significant difference (Table 5). The average length of N2 addition was also greater in the HH repertoire (6.03 nt) than CB (5.08 nt), and there was no evident difference in the length of N1 addition (CB, 6.38 nt; HH, 6.41 nt) between the two repertoires (Table 6). The diversity of N2 addition in the HH repertoire is 3.5-fold higher than that in the CB repertoire, representing the most prominent difference among all the junctional modifications (Table 7).

**Table 5:** The occurrence of junctional modifications in the repertoires of neonates and adults.

Junctional modification	Repertoire		*p*^a^	OR	95% CI
	CB %	HH %			
3VP	15.33	14.87	<2.2E−16	0.964	(0.961, 0.968)
N1	87.9	87.35	<2.2E−16	0.951	(0.947, 0.954)
5DP	4.79	4.46	2.45E−123	0.927	(0.921, 0.933)
3DP	8.28	6.74	<2.2E−16	0.80	(0.796, 0.804)
N2	64.87	85.69	<2.2E−16	3.243	(3.232, 3.253)
5JP	6.93	8.17	<2.2E−16	1.195	(1.189, 1.201)
3VT	68.07	68.66	8.52E−79	1.028	(1.025, 1.031)
5DT	88.01	88.67	<2.2E−16	1.067	(1.062, 1.071)
3DT	80.11	83.45	<2.2E−16	1.252	(1.248, 1.256)
5JT	77.35	80.84	<2.2E−16	1.236	(1.232, 1.240)

*^a^Calculated by the logistic regression*.

**Table 6:** The average length (nt) of junctional modifications in the repertoires of neonates and adults.

Junctional modification	Repertoire		*t*^a^	*d*	*p*	95% CI
	CB	HH				
3VP	1.56 ± 0.75	1.56 ± 0.75	5.117	0.01	3.11125E−07	(0.004, 0.009)
N1	6.38 ± 4.43	6.41 ± 4.57	−10.679	−0.01	1.28016E−26	(−0.041, −0.028)
5DP	1.64 ± 0.8	1.6 ± 0.78	16.360	0.05	3.92882E−60	(0.036, 0.045)
3DP	1.51 ± 0.78	1.38 ± 0.7	74.297	0.18	<2.2E−16	(0.132, 0.139)
N2	5.08 ± 4.25	6.03 ± 4.68	−270.882	−0.21	<2.2E−16	(−0.959, −0.945)
5JP	1.43 ± 0.73	1.37 ± 0.72	31.284	0.08	1.4071E−214	(0.053, 0.061)
3VT	2.84 ± 1.99	2.75 ± 1.89	53.757	0.04	<2.2E−16	(0.083, 0.089)
5DT	6.91 ± 5.01	7.21 ± 5.12	−81.316	−0.06	<2.2E−16	(−0.302, −0.288)
3DT	5.93 ± 4.15	6.55 ± 4.46	−196.632	−0.15	<2.2E−16	(−0.632, −0.620)
5JT	4.97 ± 4.37	5.78 ± 5.02	−649.265	−0.17	<2.2E−16	(−2.251, −2.238)

*^a^Calculated by Student’s t-test*.

**Table 7:** The number of different types of P/N additions in the repertoires of neonates and adults.

Library	P/N additions					
	3VP	5DP	3DP	5JP	N1	N2
CB	67	62	65	33	466,692	213,226
HH	72	74	101	38	935,620	751,780

**Figure 5:**
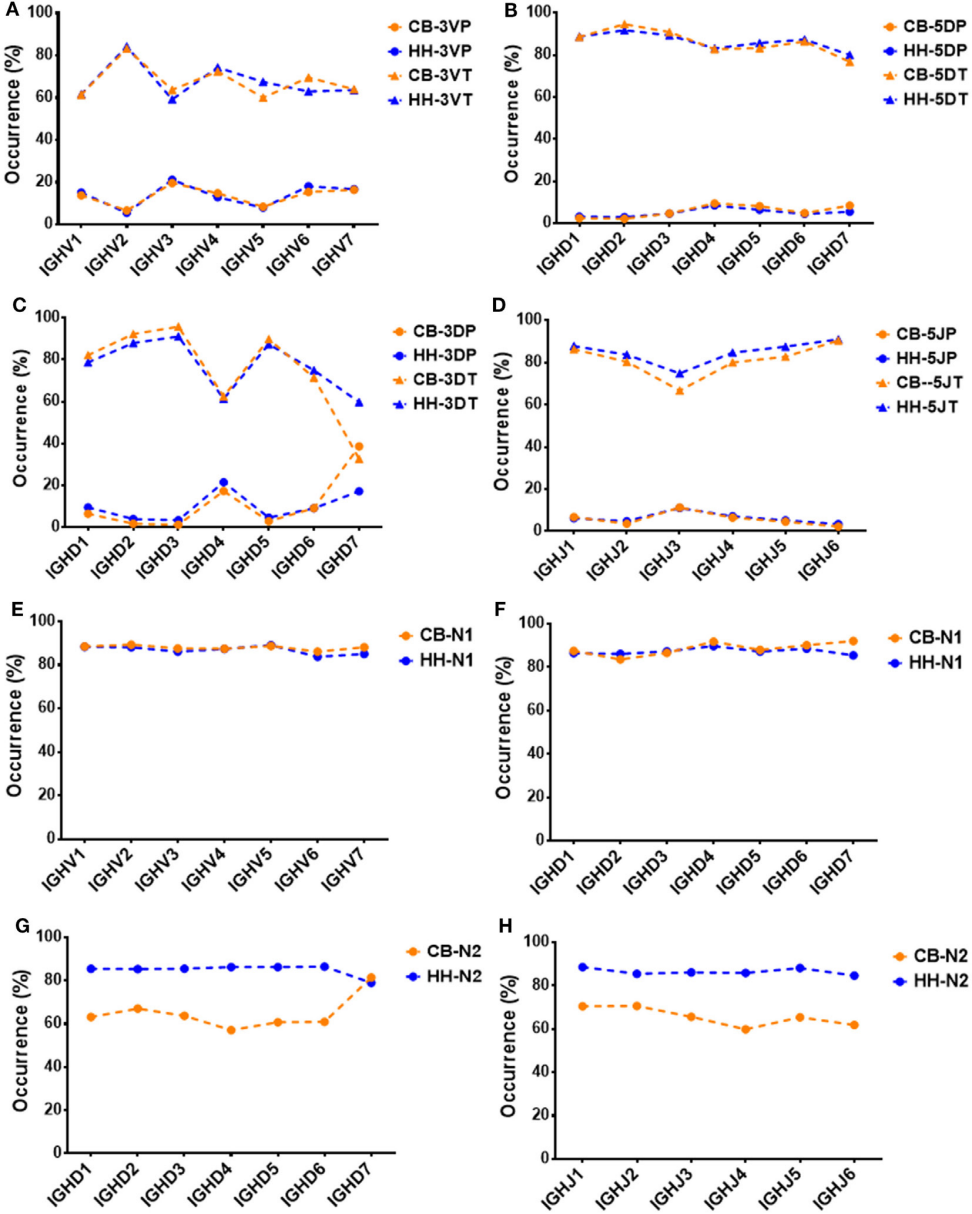
The association between VDJ gene groups and the occurrence of P, N additions or exonuclease trimmings in VDJ junctions. **(A)** The association between the occurrence of 3VP, 3VT, and IGHV gene groups. **(B)** The association between the occurrence of 5DP, 5DT, and IGHD gene groups. **(C)** The association between the occurrence of 3DP, 3DT, and IGHD gene groups. **(D)** The association between the occurrence of 5JP, 5JT, and IGHJ gene groups. **(E)** The association between the occurrence of N1 and IGHV gene groups. **(F)** The association between the occurrence of N1 and IGHD gene groups. **(G)** The association between the occurrence of N2 and IGHD gene groups. **(H)** The association between the occurrence of N2 and IGHJ gene groups.

Next, we analyzed the association between VDJ genes and the occurrence of N/P addition, along with exonuclease trimming in junctions (Figure 5). When combined the CB and HH repertoires together, most of the IGHV, IGHD, or IGHJ gene showed no or only slight statistical differences among the different subgroups, except for IGHD7, which displayed higher occurrence of 3DP and lower occurrence of 3DT than the other IGHD subgroups (Figure 5C; Table 8). Intriguingly, we found that such statistical difference was solely resulted from the CB repertoire. As shown in Figure 5C, the occurrence of the 3DP addition related to IGHD7 was evidently higher in CB as compared to the HH repertoire, while the 3DT trimming was lower. Furthermore, the N2 addition related to IGHD7 was significantly higher than that of any other IGHD subgroup in the CB repertoire, but lower than any other IGHD subgroup in the HH repertoire (Figure 5G). Except for IGHD7, all other gene subgroups had a considerably higher occurrence of N2 addition in the HH repertoire than that in the CB repertoire, and the occurrence of other types of addition or trimming (3VP, 3VT, 5DP, 5DT, 3DP, 3DT, 5JP, 5JT, and N1) did not show a significant difference between the two repertoires.

**Table 8:** The association between VDJ genes and the occurrence of N/P addition along with exonuclease trimming in junctions.

	CB			HH		
	*p*^a^	OR	95% CI	*p*	OR	95% CI
IGHV-3VP	<2.2E−16	1.062	(1.060, 1.065)	1.93E−15	0.993	(0.991, 0.995)
IGHV-3VT	<2.2E−16	1.074	(1.072, 1.076)	<2.2E−16	1.144	(1.143, 1.146)
IGHV-N1	4.90E−187	0.962	(0.960, 0.965)	7.90E−241	0.968	(0.967, 0.97)
IGHD-N1	<2.2E−16	1.102	(1.10, 1.104)	<2.2E−16	1.039	(1.038, 1.04)
IGHD-5DP	<2.2E−16	1.192	(1.189, 1.195)	<2.2E−16	1.099	(1.097, 1.101)
IGHD-5DT	<2.2E−16	0.839	(0.837, 0.84)	<2.2E−16	0.915	(0.913, 0.916)
IGHD-3DP	<2.2E−16	1.659	(1.655, 1.663)	<2.2E−16	1.138	(1.136, 1.14)
IGHD-3DT	<2.2E−16	0.642	(0.641, 0.643)	<2.2E−16	0.848	(0.847, 0.849)
IGHD-N2	<2.2E−16	1.039	(1.038, 1.041)	5.93E−56	1.01	(1.009,1.011)
IGHJ-N2	<2.2E−16	0.917	(0.915, 0.918)	<2.2E−16	0.969	(0.968, 0.971)
IGHJ-5JP	<2.2E−16	0.823	(0.820, 0.825)	<2.2E−16	0.749	(0.747, 0.751)
IGHJ-5JT	<2.2E−16	1.264	(1.262, 1.267)	<2.2E−16	1.367	(1.365, 1.369)

*^a^Calculated by the logistic regression*.

## Discussion

In this study, we adapted Illumina-based high-throughput sequencing to analyze characteristics of the IgH repertoires of the CB samples from neonates and peripheral blood samples from healthy adults. A total of 26,101,061 antibody sequences were initially obtained from 43 individuals, and a series of strict data cleaning procedures were employed to remove unproductive or biologically implausible sequences. Furthermore, we introduced a strict definition of “unique” antibody clone, which only refers to the unique antibody sequence containing a unique VDJ gene rearrangement or a unique CDR3 sequence. Although the unique VDJ gene rearrangements can represent the genetic background of antibody clones, the junctional modification occurred in the CDR3 regions serves as one of the critical mechanisms for the generation of antibody diversity. Therefore, we include the sequences containing the identical VDJ gene rearrangement but unique CDR3 sequences for the representation of junctional diversity. For the sequences with the same VDJ gene rearrangement and the identical CDR3, only one sequence can be preserved in our dataset. Under this circumstance, we may lose sequences with nucleotide polymorphisms in the IGHV, but the representativeness of our data was not affected, since more than 98% of our original sequences share at least 90% identity with the sequences from IGMT database (data not shown). Finally, a total of 10,513,005 unique antibody clones (40.3% of raw sequences) were identified. By using these procedures, we can condense the large data size and eliminate the noise of dataset, thereby facilitating the subsequent data statistics and analysis.

A couple of interesting findings were made from the comparative study of the neonatal and adult IgH repertoires. Our study confirmed the extremely large diversity of human IgH repertoires, as less than 1% of unique clones were shared by the two repertoires. Despite the difference in sequences, we found unexpectedly high levels of similarity between the two repertoires regarding the VDJ gene usage, the characteristics of CDRs, and the occurrence of certain junctional modifications. Surprisingly, the most significant difference came from the occurrence frequency of N2 addition, a process that occurs during V-D-J recombination for introducing random nucleotide additions between D- and J-gene segments. Our study also demonstrated that the IgH repertoire of healthy adults was more diverse than that of neonates, which added the evidence that the fetal repertoires were relatively limited compared to the adult repertoires. The higher occurrence of hypermutations and the N2 addition might be the reason why the repertoire of adults had higher diversity than the neonates.

The VDJ gene usage has been a topic of considerable interest because it is possible that the immune repertoires could be skewed toward a single VDJ gene family or a single VDJ gene. For the IGHV usage in our data, we found that the most frequently used IGHV gene family was IGHV3 in the neonatal repertoire and IGHV4 in the adult repertoire, and the most preferentially utilized IGHV subgroup was IGHV4-59 in both repertoires. Previous studies using 454 sequencing showed that IGHV1 group was the most predominant IGHV group in the CB IgM repertoire (26), whereas the IGHV3 group was the most populated group in the IgM repertoire of adult populations (7). For IGHJ gene usage, the IGHJ3 group was the mostly used one in both repertoires. However, previous studies showed that IGHJ4 was mostly found in CB samples and in adults’ repertoire (7, 26). For IGHD gene utilization, IGHD3 and IGHD6 groups formed almost half of the total IGHD gene usages in our data, which was also observed in the in the CB IgM repertoire (26). The preferential usage of IGHD7-27 (DQ52) in fetal samples was reported in some previous studies (27). In our data, IGHD7-27 was also frequently observed, accounting for about 10% of the neonatal repertoire; however, IGHD 7-27 only accounted for about 1% in the repertoire of adults. The reason why our results were not exactly consistent with the previous 454 sequencing-based studies of IgM repertoire in neonates or adults might be the different sequencing depth, the vast variety of the Ig repertoires, and the difference in individuals’ genetic background.

In our study, we found that the VDJ gene usage were not significantly different between the neonates and adults. We calculated the VDJ gene usage by including the information of IgH gene alleles. In total, 178 V-, 27 D-, and 13 J-gene segments were found in our study, and only two IGHV alleles were found not shared by the two repertoires. Theoretically, the frequency of each VDJ gene allele could be 0.56% in V-, 3.7% in D-, and 7.69% in J-gene if each VDJ genes were used randomly in the VDJ rearrangements. We used these theoretical values as the threshold and divided the VDJ genes into two groups that were the frequently used (FUD) genes and the rarely used (RUD) genes (Figure S3 in Supplementary Material). For V genes, there were only 47 out of 178V genes in CB as well as 36 out of 178V genes in HH whose usage were more than 0.56% that can be defined as the FUD V genes, and most of these genes (29 FUD V genes) were shared by the two repertoires. Similarly, most of the FUD D genes (7 out of 8 FUD D genes in CB and 11 in HH) and J genes (3 out of 4 FUD J genes in CB and 3 in HH) were also shared by the two repertoires. Therefore, the reason why the VDJ gene usage did not show significant difference could be that the two repertoires shared the majority of these FUD VDJ genes. However, our data also showed that the preferred VDJ genes were not exactly same between the two repertoires. This may partly due to the effects of age (antigen exposure), but we cannot exclude the influence of the individual difference. A longitudinal investigation on the same individual(s) could be more ideal to clarify this point.

The IgH CDR3 region is the most diverse component of the antibody and typically plays a critical role in defining the specificity of antibodies (28–30). In our data, the CDR3 in the repertoire of adults were much more diverse than the neonates, but the aa usage was similar between the two repertoires. Interestingly, we found that the major difference stems from the length of CDR3 regions. The length of CDR1 or CDR2 was similar in both repertoires, since the length of CDR1 and CDR2 was mainly determined by the IGHV genes whose length diversity was restricted. In contrast, the adult repertoire displayed higher frequencies of CDR3 with 15 aa or longer, and lower frequencies of 14 aa or shorter (Figure 3C), resulting in a longer CDR3 in average in the adult repertoire (15.5 aa) as compared to the neonatal repertoire (14.5 aa), which were also observed when the dataset was divided into un-mutated and mutated sequences (Figure S2 and Table S3 in Supplementary Material). Despite this, we found that the majority of CDR3 length ranged from 10 to 20 aa in both repertoires (CB, 90.72%; HH, 86.43%), and antibodies with CDR3 longer than 20 aa only accounted for a small proportion in the two repertoires (CB, 4.92%; HH, 7.84%). Some previous studies suggested that the length of the HCDR3 sequences from the fetal repertoire were considerably shorter because of the preferred utilization of the shortest D gene, IGHD7-27 (10, 13, 27, 29, 31). Indeed, we also found that the IgH repertoire of neonates had higher usage of IGHD7-27 gene than the adults, but such effects could be compromised by the fact that the neonatal repertoire also exhibited increased occurrence of N/P additions and the smaller degree of exonuclease trimming in IGHD7-27 gene. The underlying mechanism for this phenomenon requires further investigation.

The long HCDR3 loops have previously shown to be associated with antibody auto-reactivity and poly-reactivity that can be removed from the human repertoire during B-cell development (32–34). Indeed, our data suggested that the most antibodies in human IgH repertoires had the proper length of CDR3 loops but also retained a small proportion containing long CDR3 loops. A proper CDR3 length would be necessary to the survival and mature of B cells, including proper and efficient protein folding, proper pairing with the surrogate light chain to generate a functional antibody, and finally the ability to overcome negative selection of auto-reactive receptors. However, the retention of some longer CDR3 loops would be expected to increase the repertoire diversity and facilitate binding to recessed epitopes in pathogens or the active sites of enzymes (35–38).

The junctional diversification plays an important role in expanding the diversity of CDR3. Some previous studies also described the characteristics of the junctional modifications in CDR3 regions. For instance, by analyzing hundreds of productive and nonproductive VDJ rearrangements, Souto-Carneiro et al. found that the average length and occurrence of N2 insertions of fetal, preterm, and full-term neonates were significantly less than that of the adult rearrangements in the productive B-cell repertoires. The mean length of N1, 3DT, and 5JT was also less in the neonatal productive repertoires than that of adults (39). Our study showed the similar characteristics of N2 addition, and we found that the N2 addition related to IGHD7 was significantly higher than that of any other IGHD subgroups in the neonatal repertoire, but lower than any other IGHD subgroups in the adult repertoire. In another study, the mean length of N addition and 5JT trimming was also observed to be longer in human adults than fetus (15.2 ± 0.8 vs. 8.8 ± 0.6, 7.4 ± 1.3 vs. 3.9 ± 0.9, respectively), but the nucleotides loss due to 5DT was greater in human fetus than in human adults (10.2 ± 1.1 vs. 6.2 ± 1.3) (40). By performing high-throughput 454 sequencing and IMGT/HighV-QUEST analysis of 28,169 antibody heavy chain sequences from two babies, Prabakaran et al. found that N addition (93%) and exonuclease trimming (97%) had very high occurrence rates as compared to that of P additions (26%) (26). In addition, using immunodeficient mice reconstituted with human B-cell progenitors, Kolar et al. found that the N addition of sIgM^−^ cells was longer in adult chimeric mice than the fetal and CB chimeras, and the fetal chimeras had less N2 addition in comparison with adult chimeras. The N addition of sIgM^+^ cells was slightly longer in CB chimeras than the fetal and adults (15). To compare with the previous findings, we re-calculated the occurrence and mean length of the total N addition by adding the N1 additions to the N2 of each sequences. The data suggested that the occurrence and mean length of total N addition had no significant difference between the neonates and the adults (occurrence: 88.51% in CB, 87.91% in HH; mean length: 6.48 ± 4.46 in CB, 6.48 ± 4.59 in HH), which was due to the abundant N1 additions in both repertoires that may cover the difference in N2 insertions.

Besides, a number of studies analyzed the repertoires of B-cell subpopulations and described the characteristics of VDJ usage, CDR3 length, junctional modification and somatic mutation in different B-cell subsets. The IGHV3 and IGHJ4 families were often found to be the most commonly observed gene families in the previous studies, although different grouping standards were used to divide the B-cell populations, and the usage of the VDJ gene usually showed some difference among the B-cells subpopulations (41–45). However, in our adult repertoire, IGHV4, IGHD3, and IGHJ3 were the most observe genes. It was surprisingly to find that the CDR3 lengths of IgD^+^CD27^+^ memory B cells were shorter than that of IgD^+^CD27^−^ naïve B cells, and a slight reduction in CDRH3 length was also observed in antigen-experienced repertoires compared with naive repertoires (41, 43, 45–47). Besides, lower occurrence and shorter length of N addition, as well as higher occurrence and longer length of exonuclease trimming was observed in memory B cell population (41, 44). The higher affinity antigen-experienced B cells were considered to harbor the shorter CDR3 (44, 48–50). It is noteworthy to point out that we did not discriminate different cell populations, but rather pooled B cells and extracted all the IgM antibody gene by using the specific IGHM constant region primers. Therefore, our study represents a large sample surveying of the IgM repertoires of the neonates and the adults.

In this study, we were able to achieve much deeper sequencing depth with Illumina sequencing than that of 454 pyrosequencing. Importantly, we found that most of the characteristics in the repertoires of neonates and adults were similar, but the adults possess much higher occurrence of N2 addition, which may play important role in the age-related antibody repertoire changes. Taken together, the results suggested that the major source of diversity arose from the CDR3 region, and that the junctional modulations could be one of the major determinants for the increased diversity in the healthy adults, highlighting the importance of VDJ junctional modifications, especially the N2 addition.

In-depth analyses of the IgM repertoires could not only lead to a better understanding of the components in the human humoral immune system, but also have potential practical value for the development of antibody therapeutics and vaccines. For example, previous studies suggested that bioinformatics analysis can be used to identify potentially effective antibodies similar to a targeted functional antibody by analyzing the sequenced antibody repertoire adapting a Phylogeny-based method (1, 4). Therefore, our sequence data could serve as a large database to search for potentially effective antibodies. Indeed, panels of potent human monoclonal antibodies against various disease targets have been identified recently that had no or very few somatic mutations (51–56). Additionally, with the awareness of the importance of N2 junctional motif in the antibody heavy chain, it is possible to achieve more effective antibody affinity maturation by diversifying N2 junctions inside CDR3, instead of introducing extensive somatic mutations throughout the entire antibody heavy chain. Moreover, the structural analysis of antigen-antibody complexes in repertoire-scale could be facilitated by bioinformatics methods such as Pyrosetta or Rosetta Antibody (46). These information may guide the design of vaccine candidates able to induce antibodies encoded by the most frequently used VDJ rearrangements in an individual, paving the way to the development of personalized vaccination.

## Ethics Statement

The cord blood samples were provided by NDRI (Philadelphia, PA, USA) with approval of institutional research board and donor consent. Procedures followed in this study were in accordance with the ethical standards of concerned institutional policies and the Research Donor Program of National Cancer Institute.

## Author Contributions

TY, DD, and YWE conceived and designed the project. BH, YWU, WL, and XW carried out the experiments. BH analyzed the data. TY, BH, and SJ wrote the paper with input from all co-authors.

## Conflict of Interest Statement

The authors declare that the research was conducted in the absence of any commercial or financial relationships that could be construed as a potential conflict of interest.
