# Supplementary material for: In-Depth Analysis of Human Neonatal and Adult IgM Antibody Repertoires
Source: Front Immunol. 2018 Feb 5;9:128. doi: 10.3389/fimmu.2018.00128 (PMC5807330; doi:10.3389/fimmu.2018.00128)
Supplement: Supplementary file 1 [file Image_1.PDF]

Figure S1

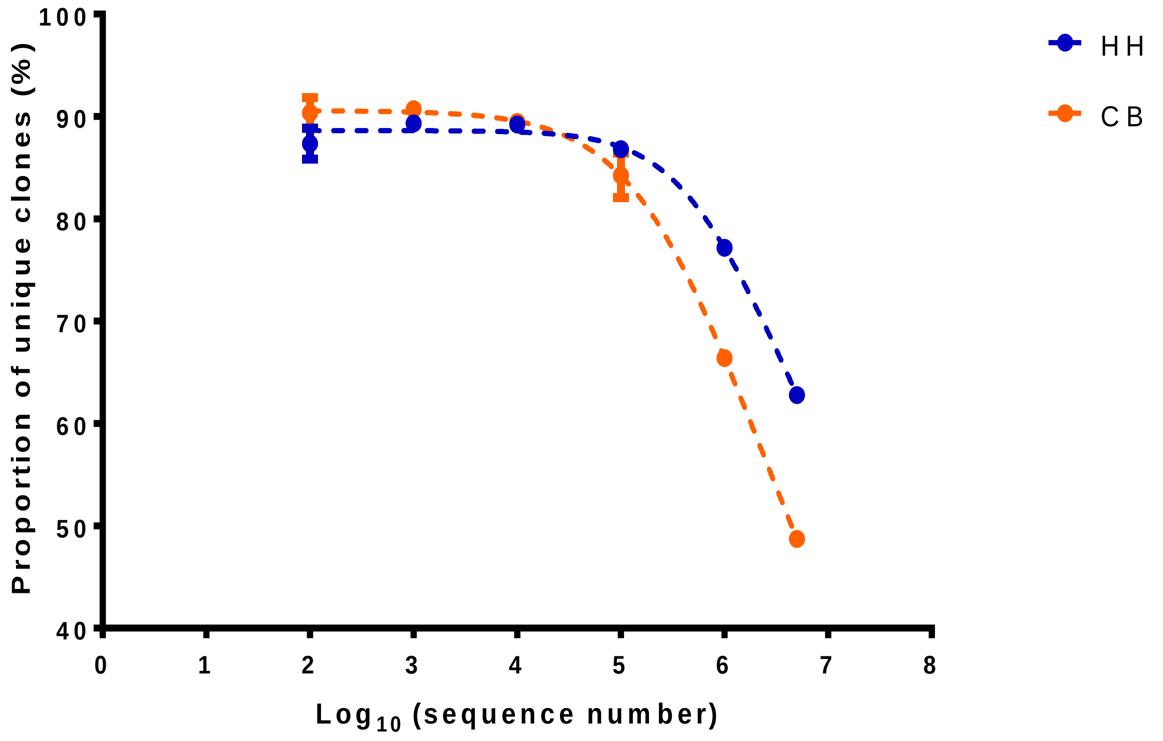

Figure S1.The diversity of the repertoires of neonates and adults. X-axis: the log10 value of the number of randomly selected sequences (100, 1000, 10000, 100000, 1000000, 50000000 sequences); Y-axis: the proportion of the unique clones out of the selected sequences. The unique clone was referred to a unique antibody sequence containing a unique VDJ gene rearrangement or a unique CDR3 sequence.

Figure S2

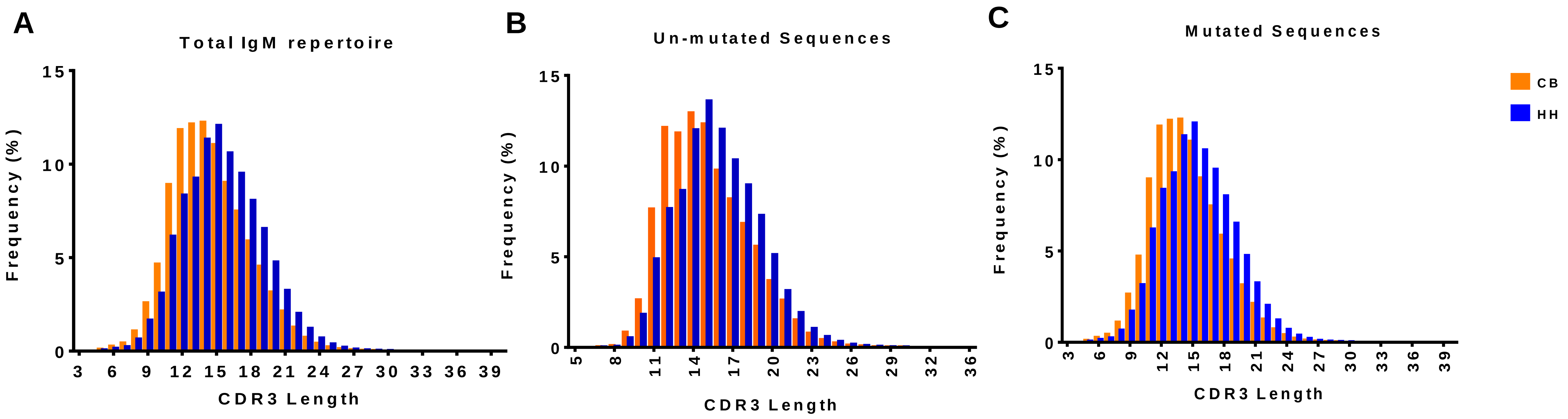

Figure S2. The CDR3 length distribution. A: The CDR3 length distribution of total IgM repertoire; B: The CDR3 length distribution of the un-mutated sequences with 100% germline identity VJ genes; C: The CDR3 length distribution of the mutated sequences.

Figure S3

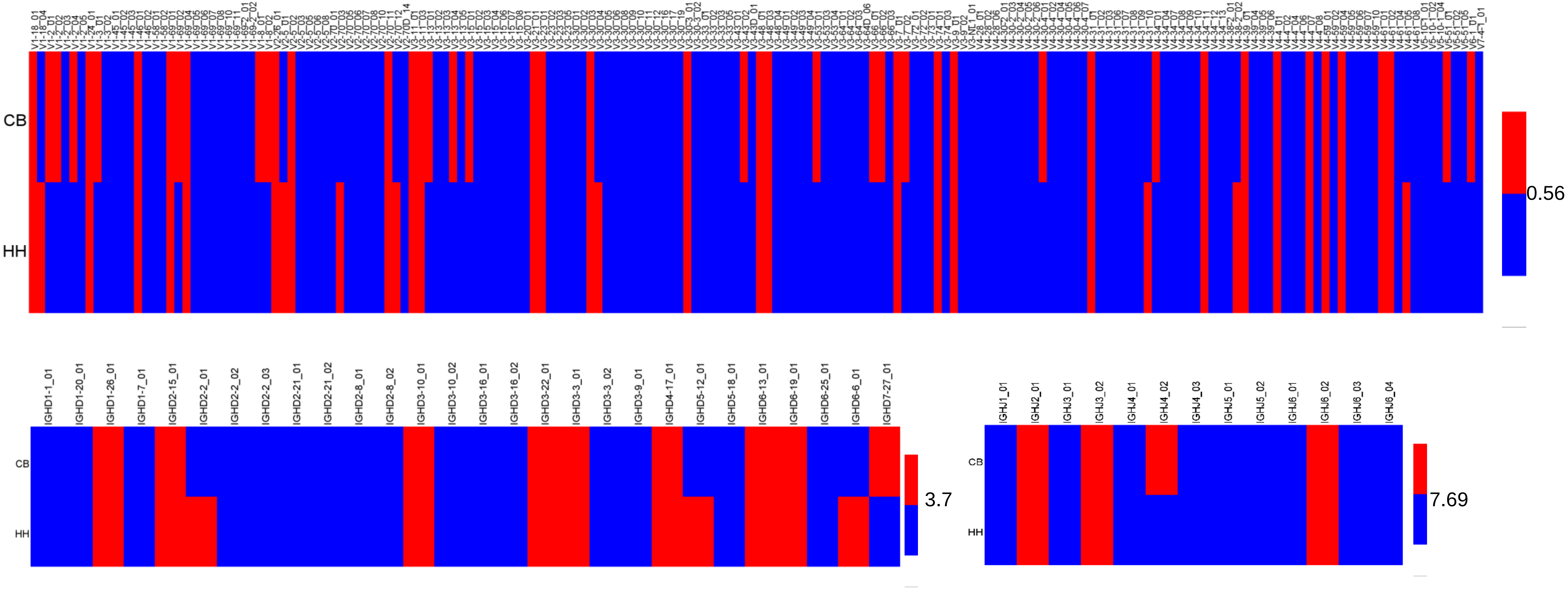

Figure S3. The usage of the VDJ genes including the gene alleles. The threshold was calculated by theoretical value when each VDJ genes were assumed to be used randomly. Totally, 178 V-, 27 D-, and 13 J-gene segments found in our study and the usage of each VDJ gene could be 0.56% in V-, 3.7% in D-, and 7.69% in J-gene theoretically.

Materials and Methods

Inclusion Criteria of the Study Population

The mothers and neonates

The mothers in their prenatal phase underwent a prenatal exam including a thorough medical, family and social history to obtain all the necessary information. The mothers before pregnancy should meet the inclusion criteria as the healthy adults (below). A regular physical exam should be included to evaluate the fetal growth in uterus and check whether the mother have any signs of cancer or infections or any recent exposures to things which might harm the baby. Especially, the mothers should not have infections that can also infect the fetus, including rubella, malaria, T. gondii, syphilis, hepatitis, herpes zoster, and HSV.

A brief screening examination of the neonates should be conducted to check the face, eyes, mouth, chest, abdomen, spine and limbs in order to exclude major abnormalities. Importantly, the neonates should not have any signs and symptoms of infection or have risk factors that predispose them to infection, including low birth weight, premature or prolonged rupture of maternal membranes, septic or traumatic delivery, fetal anoxia and maternal peripartum infection.

The adults

The healthy adults included in our study have been demonstrated with no signs and symptoms of infection one month before blood sampling, and underwent a routine health check with no history of smoking, alcohol and drug abuse and with no history of known major diseases, like cancer, autoimmune diseases, endocrine and

metabolic disorders, etc.

Table S1. The Number of Lymphocytes and B Lymphocytes

ID	Cord blood		Adult		Reference
	Total lymphocytes	B lymphocytes	Total lymphocytes	B lymphocytes	
1[a]	4.9 (3.5–6.3)	0.8 (0.3–1.0)	2.1 (1.1–2.4)	0.2 (0.1–0.4)	de Vries E et al, 2007
2 [a,c]	34.0 (18.8–52.0)	6.5 (0.9–13.0)	2,057(1,379–3,663)	0.34(0.193–0.628)	Duchamp M et al, 2014
3 [b]	-	0.905	-	0.368	Lin et al, 1998
4 [b]	5.4(3.1–9.4)	0.54(0.14–2.0)	2.3 (1.2–4.1)	0.23 (0.064–0.82)	Schatorjé et al, 2011
5 [a]	-	0.558	-	0.677	Smet et al, 2011
6 [a]	4.42	0.486	2.263	0.24	Berrón-Ruíz et al, 2016
7 [b,c]	3.896	0.5136	2.196-2.101	0.229-0.151	Veneri et al, 2007
8 [b]	5.233	0.718	1.941	0.192	D'Arena et al, 1998
9 [a]	4.61	0.75	1.5	0.12	Walker et al, 2011
10	-	-	-	0.157	Perez-Andres et al, 2010
11 [a]	6.673	0.924	2.364	0.199	Morbach et al, 2010
12 [a]	3.8	0.9	2.1	0.2	Piątosa et al, 2010

a: medians; b: mean; c: the blood sample of neonates were not cord blood.

Reference:

1. de Vries E, de Bruin-Versteeg S, Comans-Bitter WM, de Groot R, Hop WC, Boerma GJ, Lotgering FK, et al. Neonatal blood lymphocyte subpopulations: a different perspective when using absolute counts. Biol Neonate. (2000) 77(4):230-5.
2. Duchamp M, Sterlin D, Diabate A, Uring-Lambert B, Guérin-El Khourouj V, Le Mauff B, et al. B-cell subpopulations in children: National reference values. Immun Inflamm Dis. (2014) 2(3):131-40. doi: 10.1002/iid3.26
3. Lin SC, Chou CC, Tsai MJ, Wu KH, Huang MT, Wang LH, et al. Age-related changes in blood lymphocyte subsets of Chinese children. Pediatr Allergy Immunol. (1998) 9(4):215-20.
4. Schatorjé EJ, Gemen EF, Driessen GJ, Leuvenink J, van Hout RW, van der Burg M,et al. Age-matched reference values for B-lymphocyte subpopulations and CVID classifications in children. Scand J Immunol. (2011) 74(5):502-10. doi: 10.1111/j.1365-3083.2011.02609.x.
5. Smet J, Mascart F, Schandené L. Are the reference values of B cell subpopulations used in adults for classification of common variable immunodeficiencies appropriate for children? Clin Immunol. (2011) 138(3):266-73. doi: 10.1016/j.clim.2010.12.001.
6. Berrón-Ruíz L, López-Herrera G, Ávalos-Martínez CE, Valenzuela-Ponce C, Ramírez-SanJuan E, Santoyo-Sánchez G, et al. Variations of B cell subpopulations in peripheral blood of healthy Mexican population according to age: Relevance for diagnosis of primary immunodeficiencies. Allergol Immunopathol (Madr). (2016) 44(6):571-579. doi: 10.1016/j.aller.2016.05.003.
7. Veneri D, Franchini M, Vella A, Tridente G, Semenzato G, Pizzolo G, et al. Changes of human B and B-1a peripheral blood lymphocytes with age. Hematology. (2007) 12(4):337-41.
8. D'Arena G, Musto P, Cascavilla N, Di Giorgio G, Fusilli S, Zendoli F, et al. Flow cytometric characterization of human umbilical cord blood lymphocytes: immunophenotypic features. Haematologica. (1998) 83(3):197-203.
9. Walker JC, Smolders MA, Gemen EF, Antonius TA, Leuvenink J, de Vries E. Development of lymphocyte subpopulations in preterm infants. Scand J Immunol. (2011) 73(1):53-8. doi: 10.1111/j.1365-3083.2010.02473.x.
10. Perez-Andres M, Paiva B, Nieto WG, Caraux A, Schmitz A, Almeida J, et al. Human peripheral blood B-cell compartments: a crossroad in B-cell traffic. Cytometry B Clin Cytom. 2010,78 Suppl 1:S47-60. doi: 10.1002/cyto.b.20547.
11. Morbach H, Eichhorn EM, Liese JG, Girschick HJ. Reference values for B cell subpopulations from infancy to adulthood. Clin Exp Immunol. 2010, 162(2):271-9. doi: 10.1111/j.1365-2249.2010.04206.x.
12. Piątosa B, Wolska-Kuśnierz B, Pac M, Siewiera K, Gałkowska E, Bernatowska E. B cell subsets in healthy children: reference values for evaluation of B cell maturation process in peripheral blood. Cytometry B Clin Cytom. 2010, 78(6):372-81. doi: 10.1002/cyto.b.20536.

Table S2. The primers used to construct the sequencing fragements

First Round PCR	Primers
HuIgM Reverse	5′-TGG AAG AGG CAC GTT CTT TTC TTT-3′
HuVH1B/7A-FOR	5′-GCT GCC CAA CCA GCC ATG GCC CAG RTG CAG CTG GTG CAR TCT GG-3′
HuVH1C-FOR	5′-GCT GCC CAA CCA GCC ATG GCC SAG GTC CAG CTG GTR CAG TCT GG-3′
HuVH2B- FOR	5′-GCT GCC CAA CCA GCC ATG GCC CAG RTC ACC TTG AAG GAG TCT GG-3′
HuVH3B- FOR	5′-GCT GCC CAA CCA GCC ATG GCC SAG GTG CAG CTG GTG GAG TCT GG-3′
HuVH3C- FOR	5′-GCT GCC CAA CCA GCC ATG GCC GAG GTG CAG CTG GTG GAG WCY GG-3′
HuVH4B- FOR	5′-GCT GCC CAA CCA GCC ATG GCC CAG GTG CAG CTA CAG CAG TGG GG-3′
HuVH4C- FOR	5′-GCT GCC CAA CCA GCC ATG GCC CAG STG CAG CTG CAG GAG TCS GG-3′
HuVH5B- FOR	5′-GCT GCC CAA CCA GCC ATG GCC GAR GTG CAG CTG GTG CAG TCT GG-3′
HuVH6A- FOR	5′-GCT GCC CAA CCA GCC ATG GCC CAG GTA CAG CTG CAG CAG TCA GG-3′
**Second Round PCR**	**Primers**
VH-F-1	5′-GTG AAG GTY TCC TGC-3′
VH-F-2	5′-GTG AAA RTC TCC TGY-3′
VH-F-3	5′-CTC ACR CTG ACC TGC-3′
VH-F-4	5′-CTK AGA CTC TCC TGT-3′
VH-F-5	5′-YYG AGA CTC TCC TGT-3′
VH-F-6	5′-CTG CGA CCC TCC TGT-3′
VH-F-7	5′-CTG AAA CTC TCC TGT-3′
VH-F-8	5′-CTG TCC CTC AYC TGC-3′
VH-F-9	5′-CTG AGG ATC TCC TGT-3′
VH-F-10	5′-CTC TCA CTC ACC TGT-3′
VH-F-11	5′-GTG AAG GTC TCC TAT-3′
VH-R-1	5′-GGT GAC CAG GGT GCC-3′
VH-R-2	5′-GGT GAC CAT TGT CCC-3′
VH-R-3	5′-GGT GAC CAG GGT TCC-3′
VH-R-4	5′-GGT GAC CGT GGT CCC-3′

Table S3. The average length of CDR3 regions of un-mutated and mutated clones from the repertoire of neonates and adults

**CDR**[a]	**Repertoire**		***T***[b]	***d***	***p***	***95%CI***
	CB	HH				
**Total IgM CDR3**	14.51±3.33	15.48±3.43	-432.565	0.29	<2.2E-16	(-0.978, -0.969)
**Un-mutated CDR3**	15.06±3.13	15.82±3.11	-66.316	-0.25	<2.2E-16	(-0.789, - 0.743)
**Mutated CDR3**	14.49±3.34	15.47±3.43	-425.779	-0.29	<2.2E-16	(-0.981, - 0.972)

a: CDR: the complementarity determining region

b: calculated by Student’s t test.
